# Anatomical Atlas

**Published:** 1881-02

**Authors:** 


*Anatomical Atlas.* Publishers, A. E. Wilde & Co., Cincinnati, Ohio.

We have received Parts V to XIV of this work, and find them fully equal to the preceding numbers. Forty-five Parts in all will be published, each Part containing four large plates, mostly life size, accompanied with ten Parts of Text of forty pages each, at the price of fifty cents per Part. Four Parts have been issued every month, and from the manner in which this Atlas is arranged, and the accuracy of the plates, it will be when completed, a most valuable work.

